# Altered age-related alpha and gamma prefrontal-occipital connectivity serving distinct cognitive interference variants

**DOI:** 10.1016/j.neuroimage.2023.120351

**Published:** 2023-09-01

**Authors:** Yasra Arif, Alex I. Wiesman, Nicholas Christopher-Hayes, Hannah J. Okelberry, Hallie J. Johnson, Madelyn P. Willett, Tony W. Wilson

**Affiliations:** a Institute for Human Neuroscience, Boys Town National Research Hospital, Boys Town, NE, USA; b Montreal Neurological Institute, McGill University, Montreal, QC, Canada; c Department of Pharmacology & Neuroscience, Creighton University, Omaha, NE, USA

**Keywords:** magnetoencephalography, multisource interference task, functional connectivity, aging, superadditivity

## Abstract

The presence of conflicting stimuli adversely affects behavioral outcomes, which could either be at the level of stimulus (Flanker), response (Simon), or both (Multisource). Briefly, flanker interference involves conflicting stimuli requiring selective attention, Simon interference is caused by an incongruity between the spatial location of the task-relevant stimulus and prepotent motor mapping, and multisource is combination of both. Irrespective of the variant, interference resolution necessitates cognitive control to filter irrelevant information and allocate neural resources to task-related goals. Though previously studied in healthy young adults, the direct quantification of changes in oscillatory activity serving such cognitive control and associated inter-regional interactions in healthy aging are poorly understood. Herein, we used an adapted version of the multisource interference task and magnetoencephalography to investigate age-related alterations in the neural dynamics governing both divergent and convergent cognitive interference in 78 healthy participants (age range: 20–66 years). We identified weaker alpha connectivity between bilateral visual and right dorsolateral prefrontal cortices (DLPFC) and left dorsomedial prefrontal cortices (dmPFC), as well as weaker gamma connectivity between bilateral occipital regions and the right dmPFC during flanker interference with advancing age. Further, an age-related decrease in gamma power was observed in the left cerebellum and parietal region for Simon and differential interference effects (i.e., flanker-Simon), respectively. Moreover, the superadditivity model showed decreased gamma power in the right temporoparietal junction (TPJ) with increasing age. Overall, our findings suggest age-related declines in the engagement of top-down attentional control secondary to reduced alpha and gamma coupling between prefrontal and occipital cortices.

## 1. Introduction

The presence of distracting/conflicting stimuli generally hinders task performance by interfering with the response to task-relevant stimuli. Multiple subtypes of such cognitive interference exist, including stimulus-stimulus (i.e., Flanker effect; Eriksen and Eriksen, 1974), stimulus-response (i.e., Simon effect; Simon, 1990), and a combination of both (i.e., multisource interference). Flanker interference mainly encompasses an extrinsic source of conflict (i.e., flanking stimuli) and thus necessitates the engagement of selective attention, while Simon interference refers to conflict when the spatial mapping of responses is incongruent with task-relevant stimuli. Regardless of the subtype, interference resolution requires cognitive control to filter irrelevant information and allocate neural resources to task-related goals. Several neuroimaging studies have identified the neural correlates of cognitive control during conflict processing, primarily in frontal, parietal, and occipital regions (Bush et al., 2003; Frühholz et al., 2011; Liu et al., 2004; Peterson et al., 2002; Van Veen and Carter, 2002). For example, anterior cingulate and prefrontal cortices have been repeatedly implicated in the recruitment and execution of cognitive control, respectively (Fox et al., 2006; Hampshire et al., 2010; Kerns et al., 2004; MacDonald et al., 2000; Van Veen and Carter, 2002; Verbruggen et al., 2010), whereas parietal activity is believed to facilitate the coordination of spatial mapping with the motor plan (Bisley and Goldberg, 2010; Buschman and Miller, 2007; Capotosto et al., 2009; Lynch et al., 1977; Mountcastle et al., 1975; Nee, 2021; Posner et al., 1984; Yantis et al., 2002), and visual areas are known to help boost bottom-up attentional processing (McDermott et al., 2017). Further, a recent study by Weisman et al. found that different variants of cognitive interference elicited distinct spectro-temporal neural oscillatory dynamics (Wiesman and Wilson, 2020b). Though studied in healthy young adults, the direct quantification of changes in oscillatory activity and associated inter-regional functional connectivity during both divergent and convergent cognitive interference in healthy aging adults is far less understood.

Co-occurring structural and functional changes predispose the aging brain to cognitive decline and this is especially true in the context of conflict processing, where studies have shown age-related increases in cortical atrophy within critical regions of the frontal/prefrontal cortices (Cabeza and Dennis, 2012; Nyberg et al., 2010; Reas et al., 2021; Salat et al., 2004; Solbakk et al., 2008). Such alterations may not only lead to inhibitory deficits secondary to executive dysfunction (Boutzoukas et al., 2021), but also an inefficient coupling of frontal cortices with the other task-relevant regions. For instance, a previous fMRI study suggested an inverse association between resting-state frontoparietal connectivity and distractor disinhibition in older adults compared to their younger counterparts (Campbell et al., 2012). Similar age-related changes in functional connectivity among frontal, insular, and motor regions during cognitive control were reported by a later study (Langner et al., 2015). Beyond frontal/prefrontal areas, activity in critical posterior brain regions like parietal and occipital cortices, which contribute to cognitive interference processing have also been shown to be prone to aging (Kennedy and Raz, 2009).

Several studies have attempted to parse apart discrete subtypes of cognitive interference in the aging population, but their findings remain inconsistent. For example, a near-infrared spectroscopy study by Kawai et al. showed substantial differences in age-related inhibition cost between younger and older adults in response to Simon but not flanker interference (Kawai et al., 2012). These findings were later replicated by a neuropsychological study (De Bruin and Sala, 2018). However conversely, a recent EEG study investigated behavioral and neural correlates of cognitive interference by using a factorial combination of the two types of conflict processing and their findings implied age-dependent effects on both (Scrivano and Kieffaber, 2021). Despite the inconsistency in results, all the above-mentioned studies suggest distinct neural mechanisms underlying Simon and flanker interference. However, no study to date has examined the impact of aging on the underlying spectro-temporal dynamics. Importantly, both alpha and gamma oscillations in occipital and prefrontal cortices have been shown to undergo changes in power during cognitive interference processing (Janssens et al., 2018; McDermott et al., 2017; Nombela et al., 2014; Suzuki et al., 2018; Wiesman and Wilson, 2020b), with a reduction usually seen in tasks requiring cognitive control with aging (Clements et al., 2021; ElShafei et al., 2020).

The central goal of the present study was to investigate how neural oscillatory dynamics serving divergent and convergent effects of stimulus-stimulus (flanker) and stimulus-response (Simon) cognitive interference are affected by healthy aging using magnetoencephalography (MEG). MEG is a noninvasive imaging modality that measures the minute magnetic fields (10^−15^ T) naturally emanating from active neuronal populations in the brain. Importantly, magnetic fields are not altered by intervening tissues (e.g., scalp, skull, cerebrospinal fluid), unlike the electrical activity recorded using electroencephalography (EEG), which results in a good spatial precision (~3–5 mm) along with an excellent temporal resolution (~1 ms), thus making it an unparalleled technology to quantify the dynamic interactions among spatially-as well as spectrally-defined neuronal pools (Hämäläinen et al., 1993; Wilson et al., 2016).

For the current study, we collected high-density MEG data in 78 healthy participants (age range: 20–66 years) who completed an adaptation of the multisource-interference task (MSIT; Bush et al., 2003; Bush and Shin, 2006). Based on the existing literature, our hypotheses were two-fold. First, we hypothesized that there would be distinct age-dependent changes in neural oscillatory activity serving Simon versus flanker interference (De Bruin and Sala, 2018; Kawai et al., 2012). Second, we also predicted alterations in fronto-occipital connectivity (i.e., coherence) in the alpha and gamma bands with increasing age (Avelar-Pereira et al., 2017; Zonneveld et al., 2019).

## 2. Methods

### 2.1. Participants

Seventy-eight healthy adults (16 females, 6 left-handed) with a mean age of 50 years, *SD* = 612.4 (range: 20–66 years) were enrolled in this study (Table 1). Exclusionary criteria included any medical illness affecting CNS function (e.g., HIV/AIDS, lupus), any neurological or psychiatric disorder, history of head trauma, current substance use, and the MEG laboratory’s standard exclusion criteria (e.g., ferromagnetic implants). All experimental procedures conformed to the standards set by the *Declaration of Helsinki.* The study protocol was approved by the local Institutional Review Board (IRB). A full description of the study was given to all participants, followed by written informed consent, which adhered to the guidelines set forth by the IRB.

### 2.2. MEG Experimental Paradigm

**Fig. 1.**
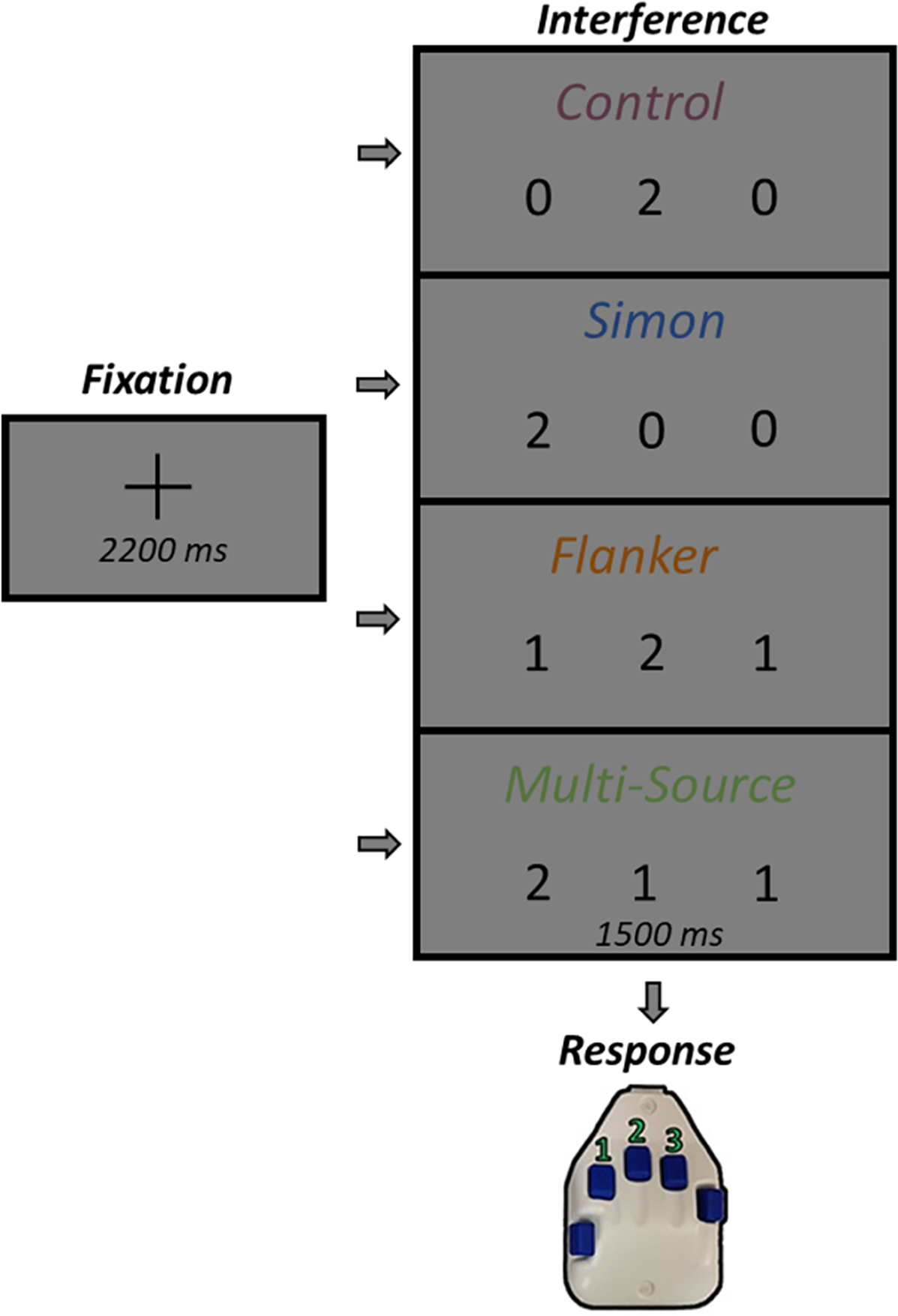
Experimental Paradigm and Behavioral performance. Each trial began with a central fixation presented for a randomly varied interstimulus interval of 2000–2400 ms. The fixation was then replaced by a vertically centered horizontal row of three equally spaced integers between 0 and 3. The presentation of the integer stimuli lasted for 1500 ms. Two of these integers were always identical (task irrelevant) and the third was different (task relevant). Prior to beginning the experiment, participants were given a five-finger button pad and instructed that the index, middle, and ring finger locations represented the integers 1, 2, and 3, respectively. Participants were then instructed that on each trial they would be presented with a horizontal row of three integers, and that the objective was to indicate the “odd-number-out” by pressing the button corresponding to its numerical identity (and not its spatial location). Using these stimuli, four interference conditions were possible: (1) control (no interference), (2) Simon (stimulus–response interference), (3) Flanker (stimulus–stimulus interference), and (4) multisource.

A modified version of the MSIT (Bush et al., 2003; Bush and Shin, 2006) was used to engage cognitive interference networks (Fig.1). Each trial began with a central fixation presented for a randomly varied interstimulus interval of 2000–2400 ms. The fixation was then replaced by a vertically centered horizontal row of three equally spaced integers between 0 and 3. These integer stimuli were presented for 1500 ms. Two of these numbers were always identical (task-irrelevant) and the third was different (task-relevant). Prior to beginning the experiment, participants were given a right-handed five-finger button pad and instructed that the index, middle, and ring finger locations represented the integers 1, 2, and 3, respectively. Participants were then instructed that on each trial, they would be presented with a horizontal row of three integers and that the objective was to indicate the “odd number-out” by pressing the button corresponding to its numerical identity (and *not* its spatial location). Speed and accuracy were also stressed to the participant at this point. Using these stimuli, four interference conditions were possible: (1) control (no interference; i.e., 1 0 0/0 2 0/0 0 3), and that the correct responses would be 1/2/3, (2) Simon (stimulus-response interference; i.e., 0 1 0/0 0 1/2 0 0/0 0 2/0 3 0/3 0 0) and the correct responses would be 1/1/2/2/3/3, (3) flanker (stimulus–stimulus interference; i.e., 1 2 2/1 3 3/1 2 1/3 2 3/1 1 3/2 2 3) and the correct responses would be 1/1/2/2/3/3, and (4) multisource (both stimulus-response and stimulus–stimulus interference; i.e., 2 1 2/3 1 3/2 2 1/3 3 1/2 1 1/2 3 3/1 1 2/3 3 2/1 3 1/2 3 2/3 1 1/3 2 2) and the correct responses would be 1/1/1/1/2/2/2/2/3/3/3/3 respectively. Trial types and responses were pseudorandomized over the course of the experiment, such that no interference condition nor any response was repeated more than twice in a row. Participants completed 100 trials of each interference condition, for a grand total of 400 trials with a thirty-second break halfway, and a total recording time of ~24 min. Custom visual stimuli were programmed in Matlab (Mathworks, Inc.) using the Psychophysics Toolbox Version 3 (Brainard and Vision, 1997) and were subtended 1.99° (horizontally) by 1.48° (vertically) degrees of visual angle and back-projected onto a semi-translucent non--ferromagnetic screen at an approximate distance of 1.07 m, using a Panasonic PT-D7700U–K model DLP projector with a refresh rate of 60 Hz and a contrast ratio of 4000:1.

### 2.3. MEG Data Acquisition

All recordings were conducted in a one-layer magnetically shielded room with active shielding engaged for environmental noise compensation. With an acquisition bandwidth of 0.1–330 Hz, neuromagnetic responses were sampled continuously at 1 kHz using a MEGIN MEG system (Helsinki, Finland) with 306 sensors, including 204 planar gradiometers and 102 magnetometers. During data acquisition, participants were monitored via real-time audio-visual feeds from inside the shielded room. Each MEG dataset was individually corrected for head motion and subjected to noise reduction using the signal space separation method with a temporal extension (Taulu and Simola, 2006).

### 2.4. Structural MRI Processing and MEG Co-registration

Prior to MEG measurement, four coils were attached to the subject’s head and localized, together with the three fiducial points and scalp surface, with a 3-D digitizer (FASTRAK 3SF0002, Polhemus Navigator Sciences, Colchester, VT, USA). Once the subjects were positioned for MEG recording, an electric current with a unique frequency label (e.g., 322 Hz) was fed to each of the coils. This induced a measurable magnetic field and allowed each coil to be localized in reference to the sensors throughout the recording session. As coil locations were also known with respect to head coordinates, all MEG measurements could be transformed into a common coordinate system. With this coordinate system, each participant’s MEG data were co-registered with their T1-weighted structural MRI prior to source space analysis using BESA MRI (Version 2.0). Structural T1-weighted MRI images were acquired using a Siemens Prisma 3-Tesla MRI scanner with a 32-channel head coil and an MP-RAGE sequence with the following parameters: TR = 2300 ms; TE = 2.98 ms; flip angle = 9°; FOV = 256 mm; slice thickness = 1 mm (no gap); voxel size = 1 × 1 × 1 mm. These data were aligned parallel to the anterior and posterior commissures and transformed into standardized space. Following source analysis (i.e., beamforming), each subject’s functional MEG images were also transformed into standardized space using the transform that was previously applied to the structural MRI volume and spatially resampled.

### 2.5. MEG Preprocessing, Time-frequency Transformation, and Sensor-Level Statistics

Eye blinks and cardiac artifacts were removed from the data using signal space projection (SSP), which was accounted for during source reconstruction (Uusitalo and Ilmoniemi, 1997). The continuous magnetic time series was divided into epochs of 1500 ms duration, with the baseline extending from −500 to 0 ms prior to the onset of the probe. Epochs containing artifacts were removed based on a fixed threshold method, supplemented with a visual inspection. In brief, for each individual, the distribution of amplitude and gradient values across all trials were computed, and those trials containing the highest amplitude and/or gradient values relative to the full distribution were rejected by selecting a threshold that excluded extreme values. Importantly, these thresholds were set individually for each participant, as inter-individual differences in variables such as head size and proximity to the sensors strongly affect MEG signal amplitude. An average amplitude threshold of 925 (SD = 200.04) fT/cm and an average gradient threshold of 230.77 (SD = 101.28) fT/(cm*ms) was used to reject artifacts. Across the group, an average of 348.35 (SD=31.78) trials per participant were used for further analysis. To ensure there were no systematic differences in the number of trials per participant, an ANCOVA was ran and this showed no significant main effect of condition, age, or the interaction, all *p’s* > .05.

Artifact-free epochs (−500 to 1000 ms, with zero defined as visual stimulus onset) were transformed into the time-frequency domain using complex demodulation (Hoechstetter et al., 2004), with a time/frequency resolution of 2 Hz/25 ms and a bandwidth of 4–100 Hz. The resulting spectral power estimations per sensor were averaged over trials to generate time-frequency plots of mean spectral density. These sensor-level data were normalized per time-frequency bin using the respective bin’s baseline power, which was calculated as the mean power during the −500 to 0 ms baseline period. The specific time-frequency windows used for source reconstruction were determined by statistical analysis of the sensor-level spectrograms across all participants using the entire array of 204 gradiometers. Briefly, each data point in the spectrogram was initially evaluated using a mass univariate approach based on the general linear model. To reduce the risk of false-positive results while maintaining reasonable sensitivity, a 2-stage procedure was followed to control for Type-1 error. In the first stage, two-tailed paired-sample t-tests against baseline were conducted on each data point, and the output spectrogram of t-values was thresholded at *p* < 0.05 to define time-frequency bins containing potentially significant oscillatory deviations across all participants. In stage two, time-frequency bins that survived the threshold were clustered with temporally and/or spectrally neighboring bins that were also above the threshold (*p* < 0.05), and a cluster value was derived by summing the t-values of all data points in the cluster. Nonparametric permutation testing was then used to derive a distribution of cluster values, and the significance level of the observed clusters (from stage 1) were tested directly using this distribution (Ernst, 2004; Maris and Oostenveld, 2007). For each comparison, 1000 permutations were computed. Based on these analyses, the time-frequency windows that contained significant oscillatory events across all participants and conditions were subjected to the beamforming analysis. For further details on our data processing pipeline, see (Wiesman and Wilson, 2020a). See supplementary material for MEG sensor level waveforms (Figure S1).

### 2.6. MEG Source Imaging and Statistics

Oscillatory neural responses were imaged using the dynamic imaging of coherent sources (DICS) beamformer (Groß et al., 2001), which applies spatial filters in the time-frequency domain to calculate voxel-wise source power for the entire brain volume. The single images were derived from the cross-spectral densities of all combinations of MEG gradiometers averaged over the time-frequency range of interest and the solution of the forward problem for each location on a grid specified by input voxel space. Following convention, we computed noise-normalized source power for each voxel per participant using active (i.e., task) and passive (i.e., baseline) periods of equal duration and bandwidth (Hillebrand et al., 2005) at a resolution of 4.0 × 4.0 × 4.0 mm. In addition, we computed noise-normalized coherence using a similar approach in our secondary analyses. For response power, such images are typically referred to as pseudo-t maps, with units (pseudo-t) that reflect noise-normalized power differences (i.e., active versus passive) per voxel. In contrast, coherence images reflect noise-normalized changes in connectivity from baseline levels between a prespecified seed voxel and every other voxel in the brain. MEG preprocessing and imaging used the Brain Electrical Source Analysis (BESA V7) software. To assess the neuroanatomical basis of the significant oscillatory responses identified through the sensor-level analysis, mean whole-brain maps were computed across all interference conditions and participants for the selected time-frequency windows.

To study the underlying cortico-cortical interactions, peak voxels in the averaged maps described above were used as seeds for the calculation of a coherence beamformer using the DICS approach (Groß et al., 2001). These images represent the voxel-wise coherence with the identified reference or seed voxel. For this analysis, we first performed a voxel-wise subtraction of the control condition coherence map from each of the three interference condition coherence maps for each participant per time-frequency component (Spooner et al., 2021). This produced participant-level whole-brain coherence maps for each of the Simon, flanker, and multisource interference conditions. Further, to investigate the potential for superadditivity of multisource interference on the neural coherence, the voxel-wise coherence values of the Simon and flanker interference maps were summed to produce a whole-brain map (per participant, per neural response), which was then subtracted from the multisource coherence map (Laurienti et al., 2005; Wiesman et al., 2020; Wiesman and Wilson, 2020b). Finally, to assess the impact of chronological age, these coherence maps were subjected to whole-brain voxel-wise correlation analyses, with age as the covariate of interest and whole-brain power as a covariate of no interest. A similar approach was used to evaluate age-dependent interference-related differences in the oscillatory power. To account for multiple comparisons, an initial significance threshold of *p* <0.001 was used for the identification of significant clusters in these whole-brain correlational maps, accompanied with a cluster (*k*) threshold of at least 10 contiguous voxels based on the theory of Gaussian random fields (Poline et al., 1995; Worsley et al., 1999; Worsley et al., 1996). Any values ±2.5 SD from the mean were considered an outlier and was removed.

## 3. Results

All participants successfully completed the study, but two were excluded due to poor behavioral performance (i.e., accuracy lesser than 2.5 *SD* from the mean).

### 3.1. Behavioral Effects

**Fig. 2.**
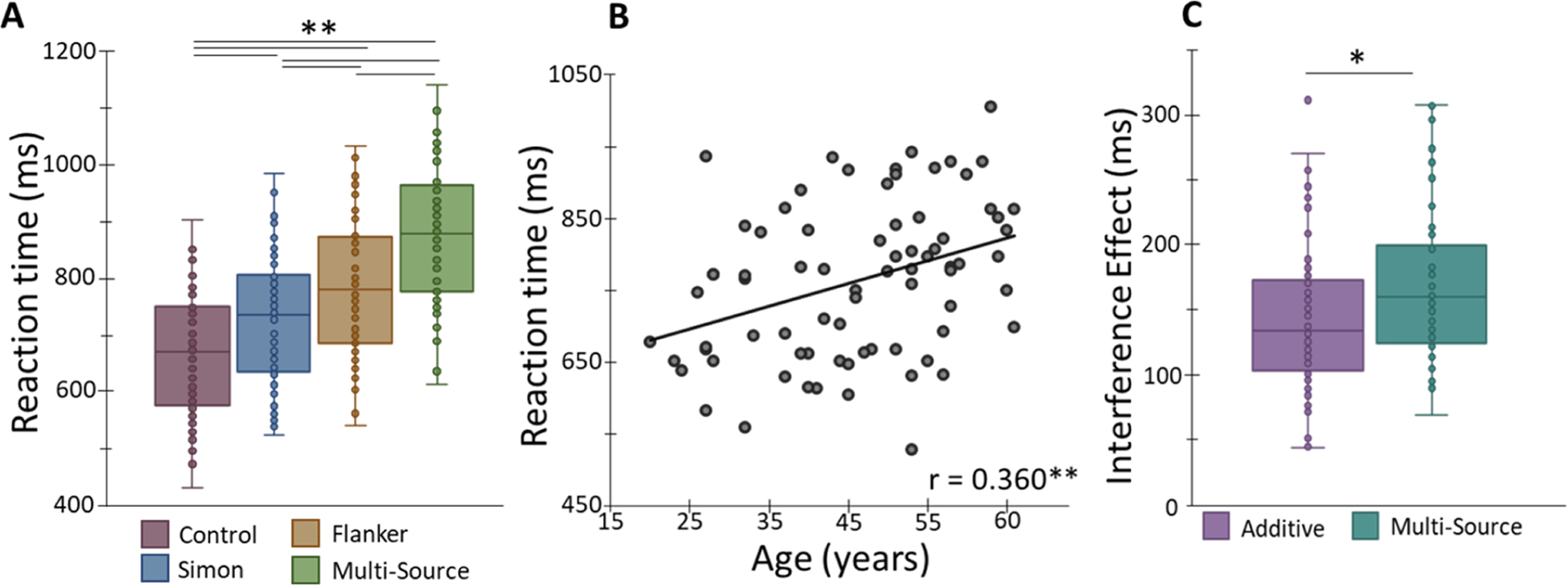
**(A)** Results from the behavioral analyses show the main effect of interference condition, with each condition differing in a stair-step pattern. Reaction time is displayed on the y-axis with interference conditions on the x-axis. **(B)** Reaction time was assessed as a function of age using Pearson correlations. There was a significant correlation between reaction time and age, such that as age increased, reaction time collapsed across all four conditions increased. **(C)** Behavioral results from the super-additivity analyses, with reaction time on y-axis and inference conditions (i.e., multisource and additive effect) on the x-axis. Plots display the individual data points, along with the median (horizontal line), first and third quartile (box), and local minima and maxima (whiskers). Error bars reflect the SEM. * *p* < .05, ** *p* =*/* < .001

A 1 × 4 ANCOVA on the behavioral data revealed significant effects of interference condition on reaction time (RT), F(3,74) = 47.91, *p* < .001 and age, F(1,74) = 10.59, *p* = .002. Post hoc comparisons showed that participants were significantly slower to respond on the Simon, *t*(75) = 22.02, *p* < 0.001, flanker, *t*(75) = 12.43, *p* < 0.001, and multisource, *t*(75) = 38.85, *p* < 0.001, conditions compared to the control trials. Furthermore, participants were significantly slower in the multisource condition than both the Simon, *t*(75) = 16.83, *p* < 0.001 and flanker, *t*(75) = 26.42, *p* < 0.001, conditions and responded significantly slower on flanker than Simon trials, *t*(75) = 9.58, *p* < 0.001 (Fig 2A).

Additionally, reaction time increased significantly with advancing age across all conditions, r = 0.360, *p* = 0.001 (Fig 2B), and the age-by-condition interaction effect was not significant, F(3, 74) = 2.57, *p* = 0.080. To probe potential age-related superadditivity effects of MSIT performance, a 1 × 3 ANCOVA comparing the effects of multisource interference and the additive model (Simon interference + flanker interference) was conducted, which showed a significant main effect of condition for RT, F(1, 74) = 5.04, *p* = 0.028. Follow-up post hoc paired t-tests showed that the concurrent presentation of the two interference sources (i.e., multisource condition) worsened the behavior, as compared to the additive effects of Simon and flanker interference in isolation, t(74) = 5.09, *p* < 0.01 (Fig 2C). Finally, neither the main effect of age F(1, 74) = .23, *p* = 0.632, nor the interaction F(1, 74) = .94, *p* = 0.335, were significant for the super-additivity reaction time effect. Further, no main effects or interaction with accuracy were found, all *p’s* > .100. Condition-wise behavioral data can be found in Table 2.

### 3.2. MEG sensor and source level oscillatory analysis

**Fig. 3.**
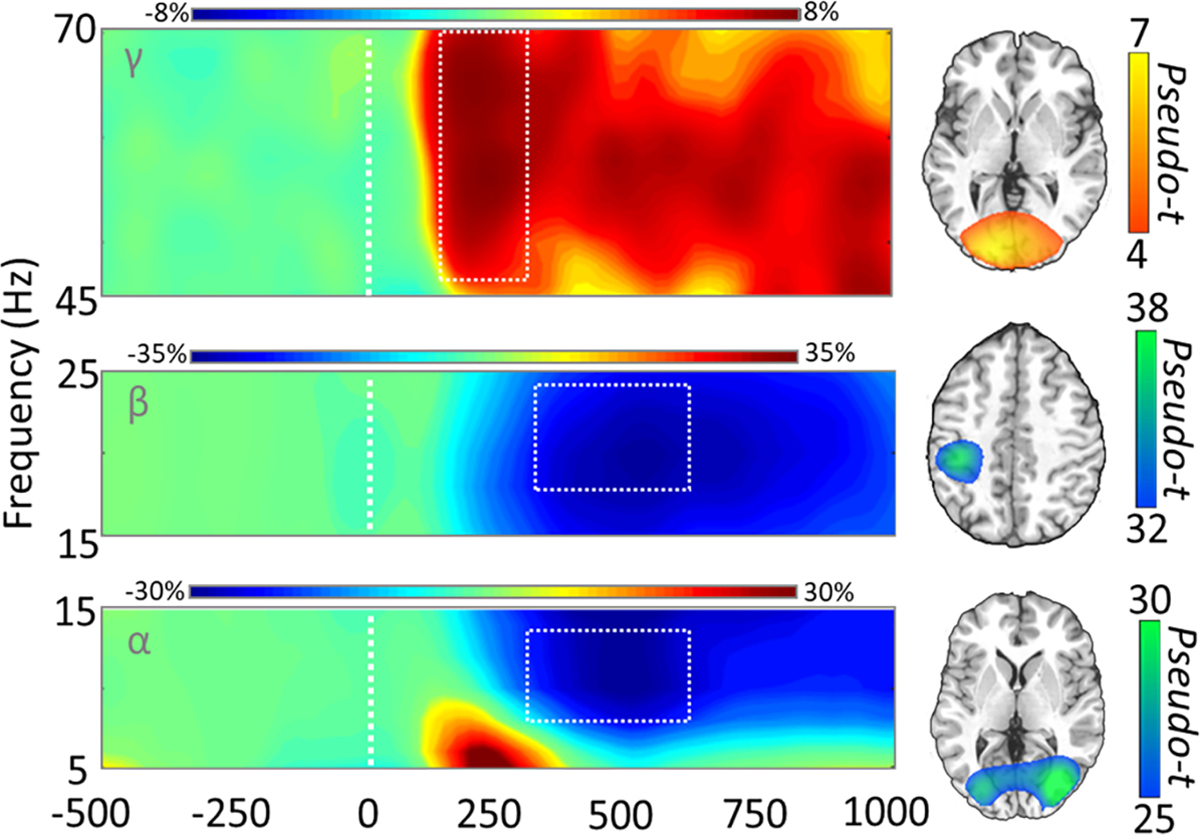
Sensor-level time-frequency analysis. The MEG sensor spectrograms (left) display the time–frequency representations of neural responses identified by cluster-based permutation analysis (see Methods section), highlighted using the white dotted boundaries. Time (in ms) is denoted on the *x*-axis and frequency (in Hz) on the *y*-axis, with the dashed white line at 0 ms indicating the onset of the integer stimuli. The color scale bar for percent change from baseline is displayed above each plot. Each spectrogram represents group- and condition-averaged data from one gradiometer sensor that was representative of the neural response in sensors over either occipito-parietal (top and bottom) or somato-motor (middle) regions. On the far right is the source-imaged representation of each response, with the color scale bar to the right denoting response amplitude in pseudo-*t* units.

After transforming the data into time-frequency space, we observed robust activity in the alpha, beta, and gamma bands (Fig 3). Specifically relative to the baseline, a significant difference (i.e., decrease or desynchronization in the active period was found to be in the alpha band at 8–14 Hz from 300 to 600 ms, *p* < .001, corrected), and this effect was predominantly observed across parieto-occipital sensors, while a significant difference (i.e., strong increase or synchronization in the gamma band 46–70 Hz; 150–300 ms, *p* <.001, corrected) was found in posterior occipital sensors. Finally, a slightly later response in the beta band (18–24 Hz; 325–600 ms, *p* < .001, corrected) was found in the left somato-motor sensors.

The time-frequency windows of interest were then imaged using a beamformer and the resulting maps per response were averaged over all participants and conditions. Source imaging of these time-frequency windows revealed that the alpha and gamma responses were originating bilaterally from lateral occipital and primary visual regions, respectively (Fig 3). In contrast, the later beta response was found to originate from the hand-knob region of the precentral gyrus, suggesting this response likely reflects the motor components of the behavioral response. Since somato-motor processing of cognitive conflict was not the focus of the current study, the beta response was not investigated in subsequent whole-brain statistical analyses.

### 3.4. Alpha and Gamma cortico-cortical coherence mapping

To evaluate age-related changes in network connectivity underlying the three types of cognitive interference, the peak voxels in alpha and gamma occipital cortices (i.e., left, and right) from the grand averaged maps mentioned above were used as the seeds for whole-brain voxel-wise cortico-cortical coherence analyses. Coherence maps were computed for each condition individually and then the control condition was subtracted from each interference condition per participant to generate coherence maps specific to the type of interference, which were then subjected to whole-brain voxel-wise correlation analyses, with age as the covariate of interest. Importantly, since both left and right occipital cortices showed similar connectivity results, the seed regions were collapsed across both hemispheres.

**Fig. 4.**
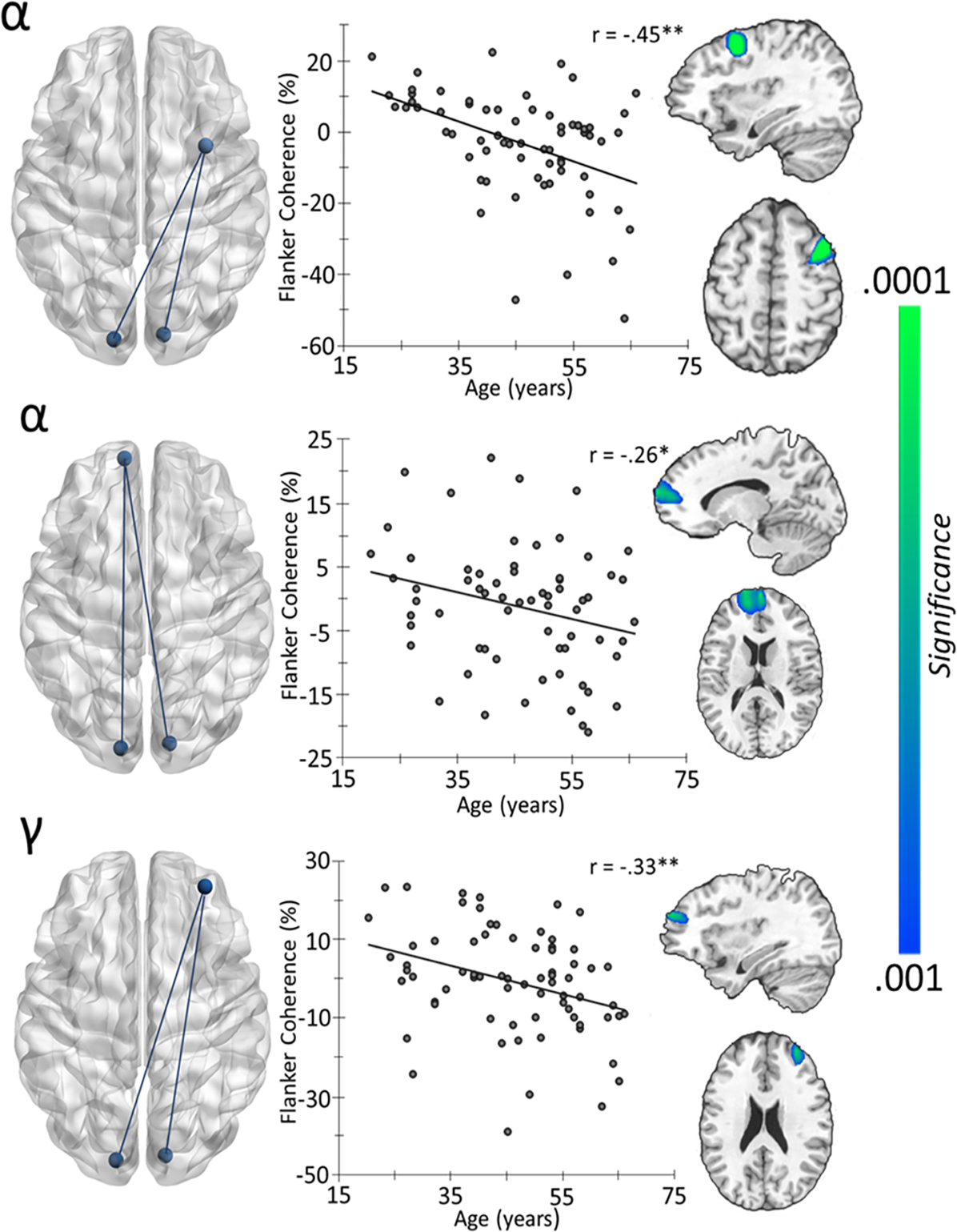
Age-related changes in cortico-cortical coherence with bilateral occipital cortices. In the flanker interference condition (flanker-control), coherence maps revealed decreased connectivity between the bilateral occipital seed and right DLPFC (top) as well as left dmPFC (middle) relative to the baseline with increasing age. In addition, weaker coherence between bilateral occipital seeds and the right dmPFC was also observed for the gamma flanker interference effect (bottom).

**Fig. 5.**
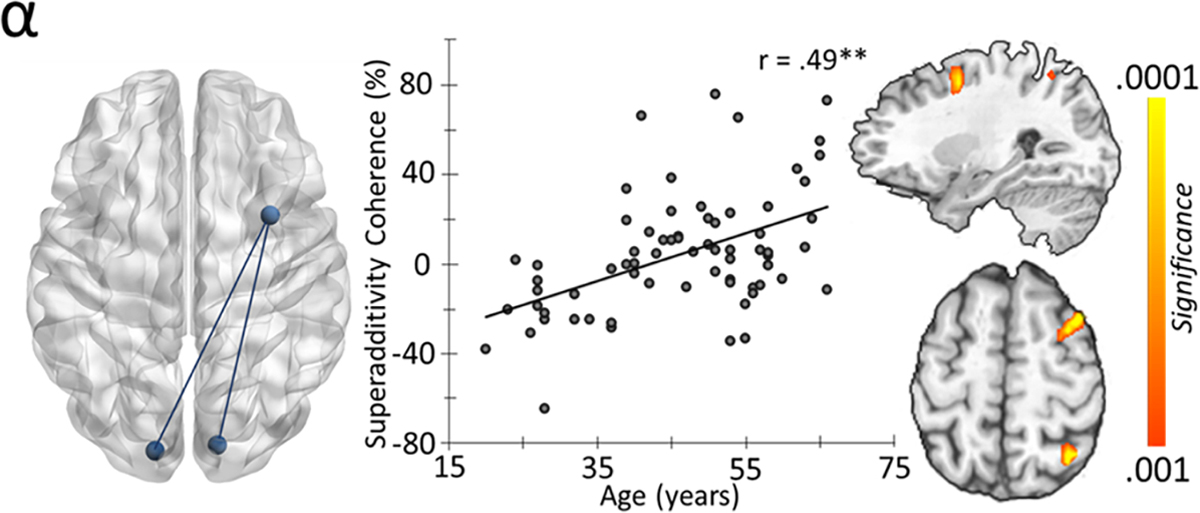
Age-related superadditivity effects on alpha prefrontal-occipital coherence. The superadditive effect on coherence was computed by subtracting the additive model (i.e., flanker + simon) from multisource interference. The effect of aging was then examine using whole-brain correlations, which indicated a positive association such that connectivity increased as a function of age.

Interestingly, for the flanker interference condition, an age-dependent reduction in alpha connectivity between bilateral occipital regions and right dorsolateral prefrontal cortices (DLPFC), r = −0.45, *p* < .001, as well as left dorsomedial prefrontal regions (dmPFC), r = −0.26, *p* = 0.035, was observed (Fig 4). A similar age-related trajectory of connectivity was found between bilateral occipital and right dmPFC r = −0.33, *p* < 0.005, in the gamma band (Fig 4). No age-related effects were observed for Simon or multisource conditions. To investigate the impact of aging on differential interference connectivity effects (i.e., stimulus-stimulus versus stimulus-response conflict), participant-level coherence maps from the Simon condition were subtracted from the flanker condition and these were subjected to whole-brain correlations with age. However, no significant effects for alpha or gamma were detected. Finally, to evaluate the impact of aging on superadditivity, the summed coherence maps of the Simon and flanker interference conditions were subtracted from the coherence map of the multisource interference condition before subjecting them to voxel-wise whole-brain correlation. This revealed a positive alpha coherence effect between bilateral occipital cortices and the right DLPFC with age, r = 0.49, *p* < .001 (Fig 5). Of note, the second, more posterior peak visible in the figure was within 4 cm of the seed and was therefore not investigated further to avoid potential spurious connectivity measurement due to possible signal leakage (Brookes et al., 2011; Schoffelen and Gross, 2009). Importantly, to limit the impact of potential confounds of power on coherence, power was covaried out.

### 3.5. Age-related conditional differences on oscillatory responses

**Fig. 6.**
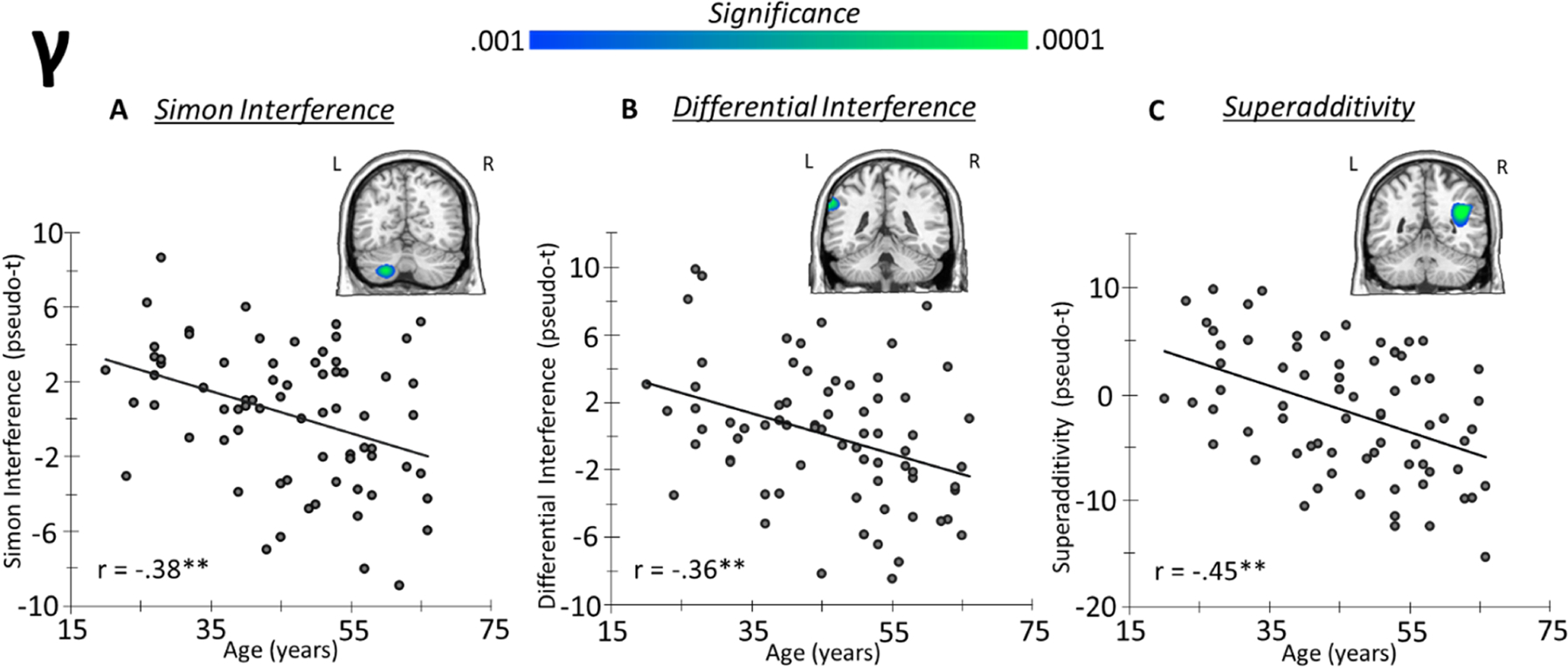
Age-related gamma oscillatory power interference effects. Whole-brain voxel-wise correlational analysis of interference maps with age revealed a decrease in gamma power in (A) left cerebellum during Simon interference (i.e., simon-control), (B) left SPL/IPS for differential interference (i.e., flanker-simon), and (C) right TPJ during superadditivity interference (i.e., multisource-additive).

To examine the impact of aging on neural oscillatory activity serving the three types of cognitive interference, subtraction interference effect maps were computed (i.e., similar to the coherence analysis) using the oscillatory power maps and this was followed by voxel-wise correlation with age. For the Simon interference condition, a decrease in gamma power with increasing age was observed in the left cerebellum, r = −0.38, *p* = 0.001 (Fig. 6A). No age-related effects were observed for flanker or multisource conditions. To investigate the impact of aging on differential interference effects (i.e., stimulus-stimulus versus stimulus-response conflict), participant-level Simon interference maps were subtracted from flanker interference maps and these were subjected to whole-brain correlation with age. Such analyses revealed a decrease in the gamma response difference between the two conditions in the left superior parietal lobule (SPL) extending into the intraparietal sulcus (IPS) with increasing age, r = −0.36, *p* = 0.002 (Fig. 6B). Finally, the analysis of the super-additive effect showed a similar result, such that the difference between the multisource interference and the additive model had an inverse relationship with age in the right temporoparietal junction (TPJ) gamma response, r = −0.45, *p* < 0.001 (Fig. 6C). Notably, no significant age-related findings were observed in the alpha band in any of the interference conditions, differential interference effects, or superadditive effects.

## 4. Discussion

In this study, we employed spatially resolved MEG with advanced source imaging and an adaptation of the MSIT paradigm (Wiesman and Wilson, 2020b) to investigate the effects of healthy aging on cortico-cortical functional connectivity and oscillatory power underlying distinct types of cognitive interference. Our whole-brain statistical analyses revealed reduced fronto-occipital alpha and gamma coherence with advancing age during flanker interference processing. Further, age-related decreases in gamma power were observed in the left cerebellum and parietal cortices for Simon and differential interference effects (i.e., flanker-Simon), respectively. Lastly, analyses of the superadditivity model showed increased alpha prefrontal-occipital coupling and decreased gamma power in right TPJ, with advancing age. The implications for these novel findings are discussed below.

Our behavioral findings showed that the participants became progressively slower in responding to the Simon, flanker, and multisource interference conditions. This is in accordance with a previous study, which showed a similar stair-step response pattern to the three cognitive interference subtypes, indexing that the required suppression of the heightened interference is responsible for the slower processing in flanker and multisource compared to Simon interference conditions (Wiesman and Wilson, 2020b). Likewise, the behavioral difference between flanker and multisource conditions can be explained by twice the magnitude of interference in the latter. Finally, the combined RT (i.e., collapsed across all the conditions) increased as a function of age, which likely suggests deficits in inhibitory processing as well as general age-related cognitive slowing/decrement (Chow and Nesselroade, 2004), although such slowing did not appear to be uniquely related to a specific type of interference.

Using a whole-brain correlational analyses, we found decreased alpha and gamma prefrontal-occipital coherence with advancing age during flanker interference processing. More precisely, weaker alpha connectivity with increasing age was identified between bilateral visual and right DLPFC as well as left dmPFC, while gamma connectivity showed the same trajectory but between bilateral occipital regions and right dmPFC with advancing age. Considering that prior studies have highlighted the roles of prefrontal (Friehs et al., 2020; Peters and Smith, 2020) and visual cortices (Wiesman and Wilson, 2020b) in conflict processing, our findings suggest that an intrinsic functional relationship between the two regions is crucial for resolving flanker interference. In the context of selective attention, the prefrontal cortex is largely known to exert top-down modulation of visual processing in posterior parieto-occipital regions (Brosnan and Wiegand, 2017; Paneri and Gregoriou, 2017) and such top-down modulatory control over visual cortical processes has been shown to parallel the strength of fronto-occipital connectivity (Kuo et al., 2011). Additionally, previous brain stimulation studies have reported changes in the flanker effect and thus cognitive control following DLPFC perturbation (Arif et al., 2020; Karuza et al., 2016; Spooner et al., 2020; Zmigrod et al., 2016), while dmPFC inactivation has also been linked to deficits in inhibitory control (De Wit et al., 2006).

In regard to aging, numerous studies have investigated its impact on top-down modulation, with many of these studies supporting the inhibitory deficit hypothesis (IDH) of aging (Fong et al., 2021; Gazzaley and D’esposito, 2007; Hasher, 2015; Hasher and Zacks, 1988; McDowd, 1997; McDowd and Filion, 1992). According to the IDH, age-related cognitive decline such as an inability to suppress irrelevant information (i.e., increased interference effects) stems from compromised inhibitory processing. Thus, our findings add support to the literature for IDH and, importantly, provide empirical evidence by showing age-related reductions in alpha and gamma prefrontal-occipital connectivity underlying flanker interference effects.

Another key finding of this study was the significant decrease in the power of gamma oscillatory responses in the left cerebellum as a function of increasing age during the Simon interference condition. The cerebellum is considered to be vital for spatial navigation via its interaction with the hippocampus (Rochefort et al., 2013; Tedesco et al., 2017) and is known to have a role in identifying the spatial features of target stimuli, a requisite for resolving the Simon interference effect (Mückschel et al., 2016). In addition, an fMRI study by Aisenberg and colleagues linked a decline in updating stimulus-response maps in the cerebellum to increasing age (Aisenberg et al., 2018). Taken together, our data may suggest that age-dependent alterations in interference resolution in the face of stimulus-response conflict is related to functional changes in cerebellar spatial mapping. We also identified differential gamma interference effects (i.e., flanker - Simon interference) in the left SPL/IPS with increasing age. This finding was expected given many studies have linked the parietal region to a number of cognitive subprocesses imperative for cognitive control, including the facilitation of goal-directed attention to task-related features of the visual stimulus (Corbetta et al., 2000; Corbetta and Shulman, 2002). It is possible that the reduced response strength with advancing age may reflect our previous interpretation of top-down attention modulation impairment as age advances.

Though not directly observed in the behavioral metrics, an aging effect on superadditivity was indexed by alpha prefrontal-occipital coherence and gamma power in the right TPJ. Superadditivity generally reflects amplified interference effects of flanker and Simon subtypes when presented concurrently rather than individually and is indicative of the recruitment of shared variant-independent neural resources between cognitive processes. More specifically, our findings showed that the impact of the superadditive effect on the prefrontal-occipital network increased with age and thus was more profound in older adults, which likely reflects age-related decreases in neural efficiency during complex cognitive processing. Such an interpretation also accords well with a recent EEG study showing that older adults are more prone to the superadditive effects of multiple conflict subtypes (Scrivano and Kieffaber, 2021). However, conversely, the superadditive cost of simultaneously presented interference types (multisource) was found to associate inversely with age in right TPJ gamma activity. This finding was unexpected, as the role of TPJ, though well-studied in bottom-up processing (Doesburg et al., 2016; Lew et al., 2018; Parks and Madden, 2013) is yet to be understood in interference resolution and cognitive control. Further, as of now, there is not a consensus on whether bottom-up processing alters or remains preserved with age advancement (Zhuravleva et al., 2014), and thus, this remains an area of active investigation. Despite our novel findings, this research is not without its limitations. First, the majority of our participants were males, which hindered our investigation to expand to possible sex differences with age. Further our findings are not accounted for chronic alcohol and tobacco use and future studies may further parse apart their effects. Finally, we did not investigate our precentral beta neural activity as its most likely a motor response, which is out of the scope of the current study. Nevertheless, future work on aging may examine how somato-motor processing serving interference subtypes changes with age, especially considering the known effects of healthy aging on motor control (Seidler et al., 2010).

To conclude, our data show age-related alterations in neural interference resolution at both the regional level (i.e., oscillatory power) as well as the network level during both solitary and concurrent presentations of interference variants. Further, our data significantly contribute to the ongoing debate about age-related deficits in inhibitory control in favor of the IDH. Finally, our study is the first to document weakened prefrontal-occipital connectivity underlying flanker interference effects with increasing age and potentially links these effects with inefficient top-down modulation of attentional control in healthy aging. Examining systems-level neuronal changes governing different subtypes of interference in pathological aging conditions such as Alzheimer’s disease could be interesting next steps.

## Supplementary Material

1

## Figures and Tables

**Table 1:** Demographics.

*Demographics (n = 76)*			

Mean age in years (SD)	56 (7)	*Tobacco consumption in the past year, %*	
Sex % Females	16 (21.06%)	Never	53 (69.74%)
*Handedness*		Once or twice	9 (11.84%)
% Left-handed	6 (7.89%)	Monthly	4 (5.26%)
*Race frequency, %*		Daily	9 (11.84%)
White	60 (78.95%)	Didn’t answer	1 (1.32%)
Black or African	8		
American	(10.53%)		
Asian	5 (6.57%)	*Alcohol consumption in the past year,%*	
More than one race	3 (3.95%)	Never	36 (47.37%)
*Ethnicity*, % Hispanics	5 (6.58%)	Once or Twice	24 (31.58%)
Mean years of education (SD)	16 (2.8)	Weekly	2 (2.63%)
*Federal Poverty Level (FPL),%*		Monthly	11 (14.47%)
Above 500%	38 (50%)	Daily	1 (1.32%)
Below 500%	23 (30.26%)	Didn’t answer	1 (1.32%)
Didn’t disclose	15 (19.74%)	Mean Cognition Total Composite score (SD)	108 (13.99)

**Table 2:** Behavioral Performance.

	Condition	Mean	Standard Deviation

**Reaction time (ms)**	Control	662.09	103.62
	Simon	731.01	107.14
	Flanker	784.11	117.79
	Multi-Source	877.79	118.79
**Accuracy (% correct)**	Control	91.49	6.93
	Simon	91.35	7.90
	Flanker	90.13	7.82
	Multi-Source	89.51	9.31

## Data Availability

The data used in this article will be made publicly available through the COINS framework at the completion of the study (https://coins.trendscenter.org/).
