# Supplementary material for: Altered age-related alpha and gamma prefrontal-occipital connectivity serving distinct cognitive interference variants
Source: Neuroimage. Author manuscript; Available in PMC 2023 Oct 15. (PMC10545948; doi:10.1016/j.neuroimage.2023.120351)
Supplement: 1 [file NIHMS1931534-supplement-1.docx]

**Supplementary Material: MEG Sensor-Level Waveforms**


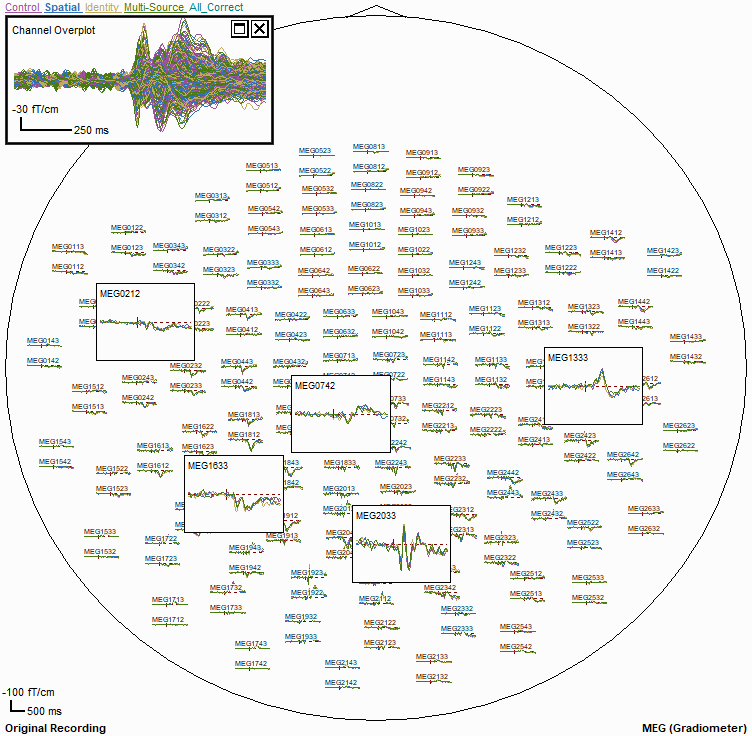

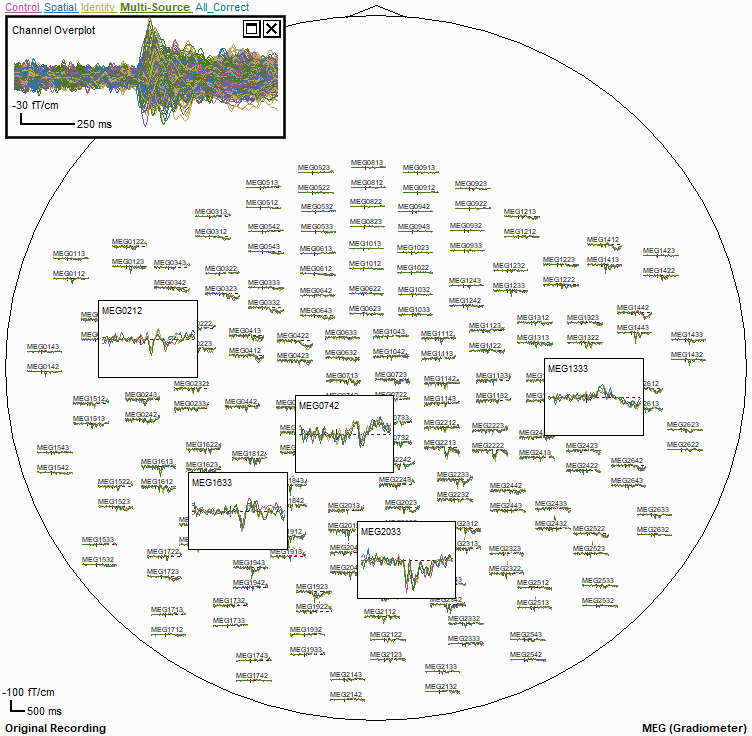


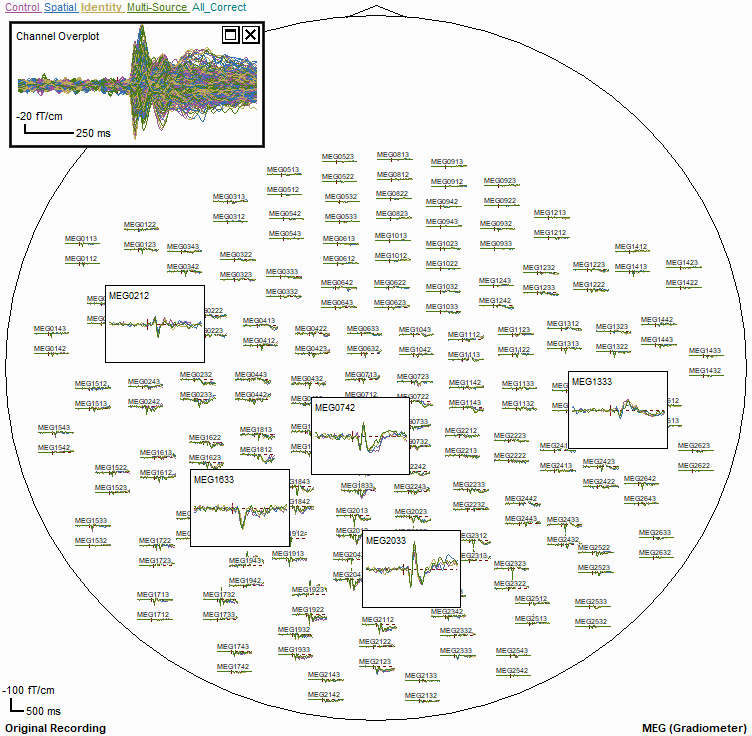


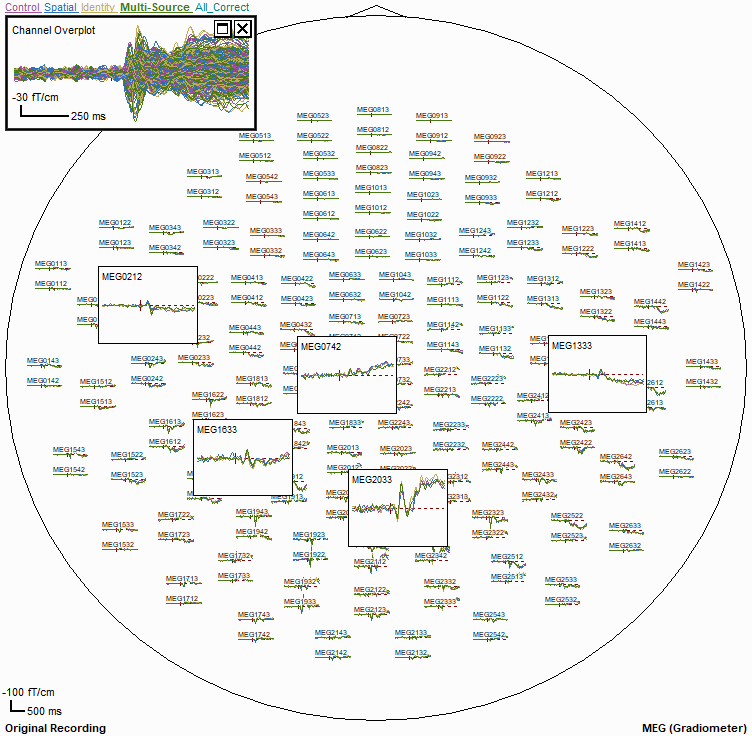


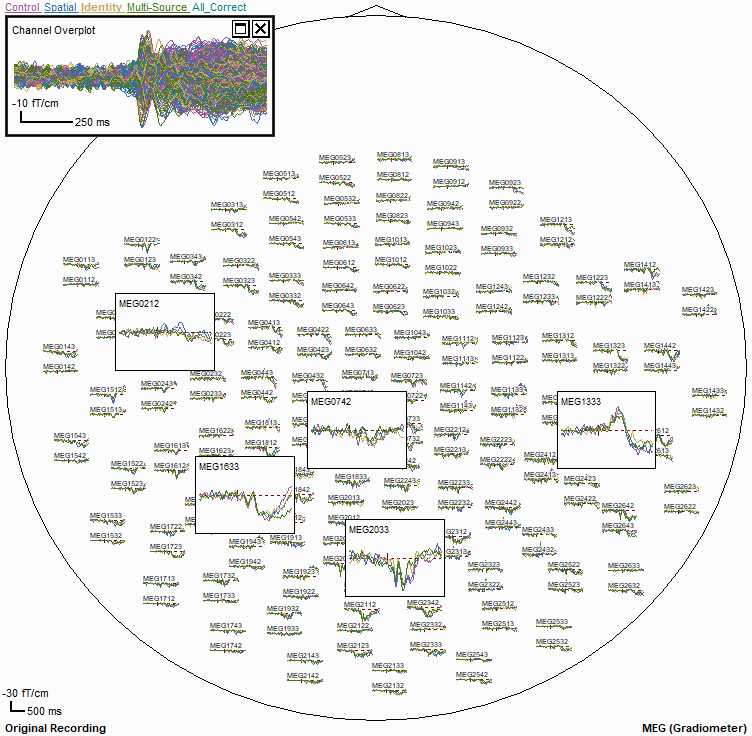


**Figure S1:** MEG sensor level waveforms of five random participants. For each, time domain averaged data from the whole array are shown, with five gradiometers enlarged to show the overall quality of data; MEG0121. MEG1633, MEG0742, MEG2033 and MEG1333. In addition, a butterfly plot (all channels overlayed) is shown in the top left corner of each participant’s panel. Colors reflect each of the four conditions.
